# Enhancing Maritime Safety: Estimating Collision Probabilities with Trajectory Prediction Boundaries Using Deep Learning Models

**DOI:** 10.3390/s25051365

**Published:** 2025-02-23

**Authors:** Robertas Jurkus, Julius Venskus, Jurgita Markevičiūtė, Povilas Treigys

**Affiliations:** 1 Institute of Data Science and Digital Technologies, Vilnius University, 01513 Vilnius, Lithuania; povilas.treigys@mif.vu.lt; 2 Institute of Applied Mathematics, Vilnius University, 01513 Vilnius, Lithuania; julius.venskus@mif.vu.lt (J.V.); jurgita.markeviciute@mif.vu.lt (J.M.)

**Keywords:** collision risk score, conformal prediction regions, long short-term memory, uncertainty quantification, vessel collision detection, vessel trajectory prediction boundaries

## Abstract

We investigate maritime accidents near Bornholm Island in the Baltic Sea, focusing on one of the most recent vessel collisions and a way to improve maritime safety as a prevention strategy. By leveraging Long Short-Term Memory autoencoders, a class of deep recurrent neural networks, this research demonstrates a unique approach to forecasting vessel trajectories and assessing collision risks. The proposed method integrates trajectory predictions with statistical techniques to construct probabilistic boundaries, including confidence intervals, prediction intervals, ellipsoidal prediction regions, and conformal prediction regions. The study introduces a collision risk score, which evaluates the likelihood of boundary overlaps as a metric for collision detection. These methods are applied to simulated test scenarios and a real-world case study involving the 2021 collision between the Scot Carrier and Karin Hoej cargo ships. The results demonstrate that CPR, a non-parametric approach, reliably forecasts collision risks with 95% confidence. The findings underscore the importance of integrating statistical uncertainty quantification with deep learning models to improve navigational decision-making and encourage a shift towards more proactive, AI/ML-enhanced maritime risk management protocols.

## 1. Introduction

In recent years, vessel trajectory prediction has garnered significant attention in the maritime industry, driven by the growing complexity of sea traffic and heightened safety concerns. However, while advances in trajectory prediction have provided valuable insights into vessel movement patterns and their forecasts, most research fails to bridge the gap between trajectory predictions and actionable safety measures. To effectively mitigate collision risks, it is essential not only to predict the potential locations of vessels but also to translate these predictions into practical measures for collision detection. This gap underscores the need for a more comprehensive approach that predicts vessel trajectories, quantifies uncertainties, and assesses collision risks. The importance of such a proactive framework is evident in the rising number of maritime incidents, and the consequences of collisions, ranging from environmental damage to financial losses and fatalities, demand urgent attention.

### 1.1. Significance of the Problem and Contribution

Traditional collision risk assessment methods, such as Closest Point of Approach (CPA) and Time to Closest Point of Approach (TCPA), rely on static, deterministic measures. While these approaches are effective in certain scenarios, they have notable limitations when applied to real-world maritime environments. CPA and TCPA focus on evaluating a single deterministic point based on the current vessel speed, heading, and position without accounting for the inherent uncertainties and variability in vessel trajectories over time. Consequently, they are less suited for dynamic and complex traffic scenarios where vessel behaviour can change due to environmental factors, human intervention, or unforeseen events. Existing models, such as heuristic or domain-based approaches, often depend on predefined parameters and lack the flexibility to adapt to evolving maritime conditions. These methods typically evaluate collision risks based on static criteria, such as predefined safe zones or ship domains, and fail to incorporate probabilistic assessments that better reflect real-world uncertainties. The key contributions revolve around the following points:

- This research employs several statistical and geometrical methods, including ellipsoidal prediction regions (EPRs), confidence intervals (CIs), prediction intervals (PIs), and conformal prediction regions (CPRs), to quantify uncertainty in vessel trajectory predictions. These methods provide a comparative analysis for determining trajectory prediction boundaries—often called guard zones, which may also be defined as ship domains—enhancing the precision of collision detection.
- The proposed methods are validated using a real-world case study, specifically the 2021 collision between the Scot Carrier and Karin Hoej cargo vessels. This validation demonstrates the practical applicability of the framework and evaluates the effectiveness of different boundary-determination techniques in detecting potential collisions.
- By training multiple models that have the same architecture, this study assesses trajectory prediction uncertainty without relying on traditional bootstrapping methods. This approach enhances the reliability of predictions by capturing variations across models, contributing to more robust boundary determination.
- A unique method is proposed to calculate and evaluate the overlapped trajectory prediction boundaries from each vessel’s perspective. This comprehensive assessment provides a collision risk score based on a Jaccard index measure, which offers a probabilistic measure of collision risk. Unlike traditional methods such as CPA, this approach considers the entire trajectory prediction boundary, holistically capturing collision risks.

Unlike traditional approaches, our method evaluates clusters of uncertainty rather than single deterministic points, allowing for a broader and more nuanced understanding of potential collision risks. This study fills a critical gap in the field, where no standardised strategy exists for vessel collision detection; even prominent Scandinavian maritime software providers continue to rely on CPA/TCPA methods. We integrate deep learning models with statistical uncertainty methods.

### 1.2. Background and State of the Art

In the context of safety and risk management, especially in maritime transportation, the terms incident and accident have distinct technical meanings. An incident is an event that poses a risk of harm but may not result in any actual damage, while an accident implies harmful outcomes such as injuries or property loss. Managing incidents is essential to preventing their escalation into accidents, reinforcing a proactive approach to safety. The Marine Accident and Incident Investigation Committee (MAIC) classifies accidents according to severity, which informs regulatory practices and safety strategies [1]. This distinction underscores the importance of early risk detection, aligning with the objective of our research to assess and mitigate potential vessel collisions before they develop into accidents.

In a comprehensive examination of marine safety over the past three decades, Polish researcher Bogalecka Magda and her colleagues provided an interesting analysis of ship accidents in the Baltic Sea [2]. Their work meticulously documents a series of specifically chosen accidents, including those involving the vessels “Dan Trimmer”, “Eagon W”, “Breant”, and “Victoria Seaways”, among others. These events have been uniformly classified as serious within the parameters set forth by the MEPC.3/Circ.3 Convention [3]. Notably, the predominant causative factor identified in nearly all these cases was severe meteorological and climatic conditions. More on the possible accidents and the situation in the Baltic Sea are investigated in the article [4].

The Baltic Sea Environment Protection Commission’s (HELCOM) published 2020 report provides a pertinent overview of maritime casualties within the region, indicating that cargo vessels were predominantly involved in such incidents; in the year 2020 alone, they constituted 51% of all reported maritime casualties, amounting to a total of 125 incidents. Additionally, passenger vessels accounted for 29% of all casualties, with 74 reports logged [5]. These statistics reflect that cargo and passenger vessels represent the bulk of maritime traffic in the Baltic Sea. A significant observation is that most collisions occurred in port activity areas as vessels navigated their approach or within the ports’ confines, contributing to 30% of all maritime accidents. Collisions have been further categorised by the nature of the entity they involve, which includes collisions with other moving vessels, static objects such as bridges, docks, or breakwaters, and a range of other causes such as being adrift, fires, containment loss, or flooding. This stratification of collision types offers a nuanced perspective on maritime accident dynamics and is integral to developing targeted safety measures and regulations.

Maritime safety research has explored various solutions, often focusing on collision avoidance, route optimisation, or abnormal behaviour detection. These approaches employ heuristic methods, dynamic programming, or machine learning techniques, such as reinforcement learning, to optimise navigation or respond to potential threats in real time. However, most studies emphasise avoiding collisions after risks are identified rather than detecting potential collisions proactively. This study takes a distinct approach by focusing on collision detection through predictive modelling, leveraging vessel trajectory predictions to assess risks probabilistically. By shifting the focus to proactive risk detection, this work aims to bridge the gap between trajectory prediction and actionable safety measures, contributing to more effective and early intervention strategies in maritime safety.

### 1.3. Introduction to the Workflow

By building on the foundation of our previous research on vessel trajectory prediction [6], this study extends its scope to address the critical task of collision detection. While trajectory prediction provides essential insights into vessel movements, its adaptation for practical risk assessment and safety applications remains under-explored. By leveraging the predictive capabilities developed in earlier work, this research focuses on integrating statistical methods to identify and quantify collision risks. The proposed workflow utilises a combination of CIs, PIs, EPRs, and CPRs to define trajectory boundaries and assess overlaps between vessels. By analysing these overlaps, we introduce a probabilistic framework for evaluating collision risk scores, advancing from deterministic trajectory predictions to actionable safety measures. This approach contrasts with the currently used CPA method, which only predicts a single point of coordinates, providing a more limited view of collision risks.

Our research aims to enhance maritime collision risk assessment by integrating trajectory prediction and statistical methods to provide a probabilistic understanding of the future positions of vessels, accounting for the uncertainties inherent in maritime navigation and allowing for the definition of potential collision boundaries; we highlight areas based on Long Short-Term Memory (LSTM) autoencoder recursive predictions. The LSTM architecture is based on real data from the Baltic Sea region from the Automatic Identification System (AIS). By combining confidence levels with these predictive methods, our models can dynamically assess collision risks and identify high-risk scenarios from the different perspectives of nearby vessels, enabling the implementation of pre-emptive safety measures.

**Figure 1:**
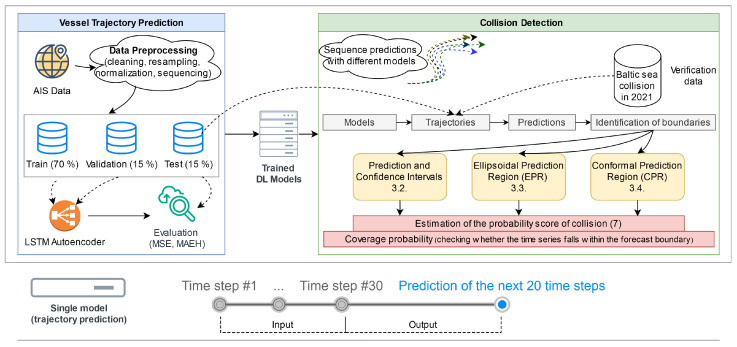
Workflow diagram of vessel trajectory prediction boundaries and collision detection.

A comprehensive workflow has been developed to provide a clear study overview (see Figure 1). The diagram illustrates the general flowchart for vessel trajectory prediction, which builds upon the results from previous studies. The primary focus of this study is utilising model predictions to define the boundaries of predicted trajectories, assessing overlapping regions with collision risk scores in real-case scenarios, and evaluating prediction accuracy using coverage probabilities. The study employs multiple trained models, each capable of producing different predictions for the same trajectory due to inherent uncertainties, allowing for the generation of diverse samples for probabilistic estimates. These methods are discussed in detail throughout the paper. Notably, each model accepts an input sequence of 30 time steps (equivalent to 30 min) and predicts the next 20 time steps (equivalent to 20 min). The process involves evaluating the predicted trajectories using a test data sample, comparing the real-time steps of the vessel’s movement against the prediction bounds, and calculating how many actual positions fall within these predicted regions. The study is validated using a historical maritime accident in the Baltic Sea in late 2021. This case study demonstrates how different methods generate distinct boundaries and how these overlapping thresholds with collision risk scores can be evaluated from the perspective of multiple vessels, highlighting the practical application of the framework in real-world collision detection scenarios.

### 1.4. Aim and Organisation of the Article

This research aims to investigate integrating deep learning algorithms to improve maritime navigational decisions and the progression of risk management techniques to enhance maritime safety. Structured comprehensively, the article begins with a literature review in Section 2, examining prior research on deep learning applications in maritime navigation to contextualise this study within the broader academic discourse. The review is organised into two subsections: Section 2.1 explores vessel trajectory prediction methods, and Section 2.2 focuses on collision risk assessment approaches. Section 3 details the methodology, outlining the data collection, model selection, and analytical techniques. Section 4 applies the methodology to both simulated and real case studies, demonstrating the predictive prowess of the models and their potential to mitigate maritime disaster risks. The article culminates in Section 5, where conclusions are drawn, highlighting the study contributions and limitations of deep learning models to maritime safety and proposing directions for future research.

## 2. Related Studies

### 2.1. Vessel Trajectory Prediction Methods and Uncertainty Estimation

In maritime navigation, the accurate prediction of vessel trajectories is paramount for avoiding collisions and ensuring safety at sea. A body of research has been dedicated to addressing this challenge, utilising a variety of sophisticated methodologies. For instance, one study by Biao Zhang and colleagues focuses on refining the precision of ship motion predictions, especially within complex environments characterised by non-stationary, non-linear, and stochastic variables [7]. This research introduces the IWOA-TCN-Attention model, a novel predictive framework that integrates deep learning networks, time-sequential data, and an attention mechanism, substantially improving the prediction accuracy.

Complementing this, another study by H. Rong approaches maritime traffic prediction using a dual lens, contemplating both ship destination and route [8]. This study distinguishes itself by its methodology of extracting vessel motion patterns from archival data, deploying multinomial logistic regression alongside Gaussian Process regression models to construct probabilistic forecasts. However, it observes that the hourly forecast increases the error and notes that additional features may hypothetically reduce this error.

The research landscape reveals a diversity of approaches for assessing predictions of vessel trajectories, notably focusing on their utility in identifying anomalous marine traffic patterns. The detection and analysis of abnormal vessel trajectories are carried out in a study by Kristoffer et al., who propose a kinematic similarity measure and even provide access to labelled data to support further research on abnormal trajectory detection [9].

LSTM neural networks have also played a significant role in enhancing the trajectory prediction accuracy. For example, Mehri employs LSTM models with a context-aware approach that integrates spatial data, such as vessel type and meteorological conditions, for data-driven movement predictions [10]. Additional research explores the broader application of LSTM networks in ship trajectory prediction, showcasing their versatility in handling maritime data [11,12,13,14,15].

Uncertainty quantification methods have been introduced to enhance the reliability of trajectory predictions J. Venskus proposed unsupervised wild bootstrapping to evaluate prediction reliability, demonstrating its effectiveness in detecting a wide array of abnormal marine traffic behaviours [16]. Additionally, abnormal vessel trajectories have been tested by creating multivariate cases of prediction intervals, assuming that the trajectory entering a defined region is considered normal.

Graphical methods for region detection, such as those proposed by Carnerero et al., utilise convex optimisation techniques to delineate prediction regions [17]. The reliability of predictions for multidimensional data is further examined using boundary estimation techniques that define regions with predefined nominal coverage rates. F. Golestaneh et al. have contributed to this field by constructing and evaluating multivariate ellipsoidal prediction regions (EPRs), delineating the uncertainty inherent in multidimensional stochastic processes [18]. While not directly applied to ship trajectory prediction, their findings offer insights into minimising the conservativeness of prediction regions.

Forecasting models often rely on statistical approaches to evaluate prediction reliability. Yin et al. analyse bus travel time forecasts using the construction of prediction intervals (PIs) [19], and Noma and Lucagbo discuss multidimensional prediction and confidence intervals (CIs) in broader contexts [20,21]. However, maritime forecasting data frequently lack a clearly defined distribution, necessitating non-parametric techniques such as Conformal Prediction Regions (CPRs). This approach, initially introduced by Shafer [22], has been applied in diverse fields, including pedestrian localisation scenarios [23], and, more recently, time-series prediction interval detection [24,25].

### 2.2. Collision Risk Assessment and Predictive Frameworks

While trajectory prediction provides the foundation for maritime safety, collision risk assessment is equally critical in ensuring safe navigation. Ship collisions pose significant threats to maritime operations due to their potential to cause substantial harm. Addressing this, Ryan Wen Liu lays out a comprehensive framework for assessing and analysing ship collision risks [26]. This framework incorporates advanced methodologies, including the quaternion ship domain and kernel density estimation. It integrates a ConvLSTM model for spatial–temporal risk prediction, marking a substantial stride in maritime collision avoidance strategies.

In expanding on collision avoidance strategies, several studies propose advanced decision-making frameworks. For instance, Xie et al. introduce a Deep Reinforcement Learning (DRL) approach to multi-vessel collision avoidance that adheres to COLREGs (Convention on the International Regulations for Preventing Collisions at Sea) [27]. Their model integrates a Collision Risk Index (CRI) into its reward function, optimising vessel behaviour in various encounter scenarios. Similarly, Seo et al. develop a CRI-based A* algorithm that balances economic route optimisation with safety considerations, effectively addressing collision avoidance routing [28].

Moreover, recent studies on unmanned aerial vehicles (UAVs) demonstrate how LiDAR–camera fusion enhances dynamic localisation and intelligent control, offering insights applicable to maritime collision avoidance [29].

Yoshioka et al. present a decision-making algorithm that utilises collision risk maps to visually represent potential risks and guide route planning, focusing on enhancing explainability for seafarers [30]. Zhou et al. focus on determining collision avoidance timing using a machine learning framework that incorporates dynamic and static factors to guide Officers on Watch (OOW) [31]. Additionally, Liu et al. propose QSD-LSTM, a novel trajectory prediction model that integrates a quaternion ship domain to enhance the prediction of vessel interactions in complex maritime environments [32].

Probabilistic models for collision risk assessment have also been explored in previous research. Mujeeb et al. assess collision risks between vessels and offshore platforms using a model based on traffic density, causation probabilities, and mitigation measures [33]. However, this approach lacks adaptability to real-time dynamic conditions. Yim et al. apply multiple linear regression to analyse the perceived collision risks based on separation distance, though their reliance on subjective evaluations limits the model’s real-time applicability [34].

Du (2020) develop a COLREG-compliant alert system for stand-on vessels that classifies conflicts into four encounter stages and nine severity levels to improve collision risk detection under dynamic conditions. However, uncertainties regarding ship domain boundaries remain a challenge in this framework [35]. Lin (2024) introduces an encoder–decoder LSTM model for regional collision risk prediction, achieving high accuracy but facing limitations due to aggregation density and clustering uncertainties [36]. Likewise, Gao (2024) employs ST-ENAGCN (Spatio-Temporal Edge-Node Attention Graph Convolutional Network) to analyse multi-ship collision avoidance scenarios. Yet, this approach may not fully address ship domain boundary uncertainties, which is crucial for reliable collision risk assessment [37].

Additional research has focused on risk evaluation frameworks. Studies such as those by Weng, Tritsarolis, and Jia delve into collision risk assessment using probabilistic and statistical methods, further highlighting the complexity of accurately predicting and mitigating maritime collision risks [38,39,40,41].

Our research aims to address the limitations highlighted in the aforementioned studies by employing a deep learning-based trajectory prediction approach that incorporates advanced uncertainty quantification methods, including EPR, CPR, and prediction intervals. These methods define trajectory boundaries and calculate overlap collision risk scores, enabling more reliable and proactive collision risk assessments in real-time maritime contexts.

By applying these predictive boundaries to real-world scenarios, such as the 2021 collision between the Scot Carrier and Karin Hoej, we demonstrate the effectiveness of deep learning models in improving maritime safety with a 95% confidence level. Our approach also integrates reliability indicators, particularly emphasising the strength of conformal prediction in accurately predicting potential collision scenarios. As the reviewed articles indicate, one specific method or its derivatives are typically used to define prediction boundaries. Therefore, this study applied various methods to specify the domain region of vessels based on predictions from deep learning models while assessing the probability score of collision risk for vessels in actual historical accidents.

## 3. Methods

In our investigation into predicting vessel trajectories, we used techniques that combine a deep learning algorithm and sophisticated statistical analysis to predict trajectories, define boundaries, and assess the awareness of collisions at sea. This section explains several key elements of our study: first, the application of deep learning to predictive modelling, followed by collision probability assessment techniques such as PI, CI, EPR, and CPR. These methods predict the ship’s further course and quantify the inherent uncertainty in these predictions.

### 3.1. Deep Learning Model for Trajectory Prediction

In collision prediction within maritime navigation, we leverage a deep learning model that employs an LSTM autoencoder architecture previously detailed in our publications [6]. The model’s strength lies in its ability to encode temporal sequences of vessel movements into a lower-dimensional latent space and reconstruct them, capturing the essential features for predicting future trajectories. The architecture comprises three key components: an encoder, a latent space, and a decoder. The encoder processes input sequences, reducing their dimensionality, while the decoder reconstructs the output from the latent representation. This specific implementation involves a sequence-to-sequence approach, where input data are flattened to transform the time steps into a singular vector that encapsulates the temporal features essential for predicting vessel paths.

For this study, the sequences are constructed from 50 time steps and split into 30 steps for the input and 20 for the output, with an average time interval of 1 min. The choice of a 20 min forecast horizon aligns with the stopping times of large vessels [42], which is documented in maritime studies, ensuring the predictions provide a sufficient lead time for assessing potential collision risks. The data used for the neural network comprise features such as encoded vessel type, geographical coordinates, speed, heading, and differences in latitude and longitude between consecutive time steps. The output sequence consists only of the coordinate features, and the input sequence consists of the aforementioned vessel characteristics. These features are valuable for understanding vessel behaviour and predicting further points along the trajectory. The parameters used in the architecture are given in Table 1.

**Figure 2:**
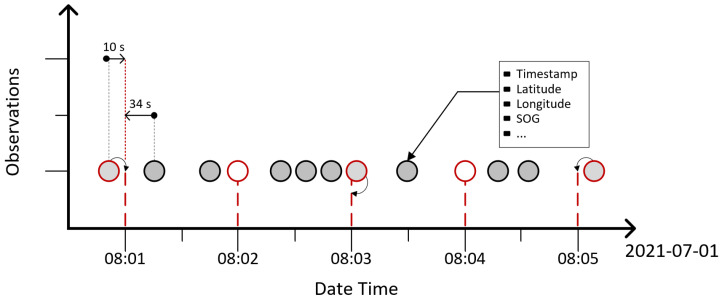
AIS data resampling based on time series. Grey dots represent all AIS observations, while red boundary circles indicate points selected using the k-nearest neighbour method for standardizing time steps.

A resampling method (see Figure 2) is applied to handle the challenges associated with AIS data, such as signal gaps and variability in reporting frequencies across different vessels. The time series data are resampled using the nearest neighbour algorithm, ensuring a standardised time step of one minute. This preprocessing step mitigates inconsistencies in the dataset, which can exhibit overlapping or missing observations despite its large semi-structured nature. Additionally, spatial filtering is applied to remove instances where large gaps exist between consecutive AIS points, preventing unrealistic trajectory jumps. This ensures that the vessel movements remain continuous and representative of real-world navigation patterns. By combining time standardisation and spatial filtering, the dataset maintains temporal consistency while reducing distortions in trajectory prediction, ultimately improving the reliability of the predictive model. It is important to note that the current model does not include meteorological or weather-related features, such as wind speed or wave height. These factors, while critical in certain scenarios, are beyond the scope of this study. Future research will focus on integrating these additional parameters to enhance the accuracy and applicability of trajectory predictions in dynamic maritime environments.

**Figure A1:**
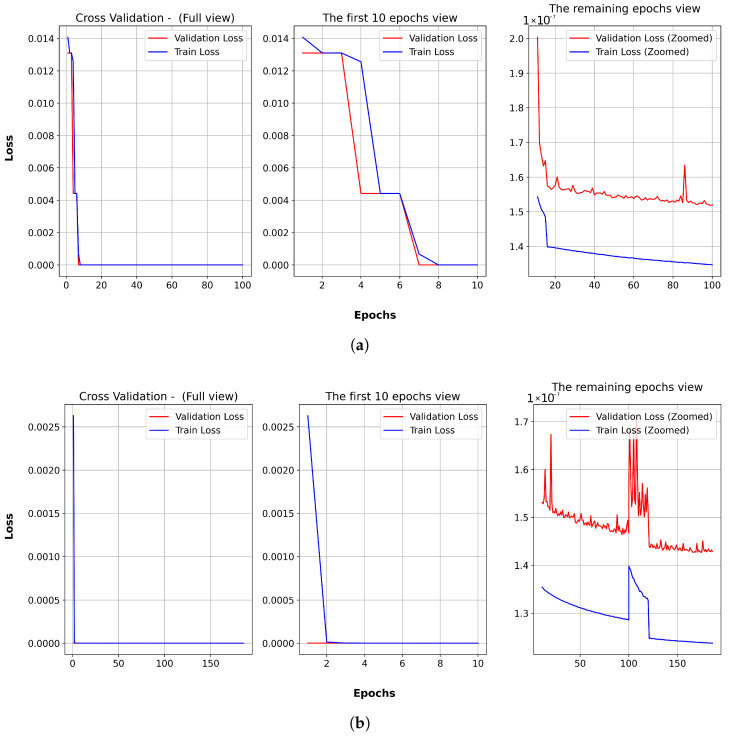
Cross-validation results for different models: (**a**) cross-validation plot for a random model; (**b**) cross-validation plot for the best model.

The model is trained, tested, and validated on a dataset split according to a 70:15:15 ratio, a common approach in scientific research [43,44] that ensures a robust validation process. This split is particularly suitable given the size of the dataset, containing almost one million sequences in the region, allowing sufficient data for each subset without over-representing or under-representing any scenario. The data are shuffled before training, ensuring the model’s exposure to diverse scenarios and enhancing its generalisation capabilities. Additionally, the cross-validation results included in Figure A1 illustrate the training process and validate the stability and convergence of the model. The remarkable point is that the study does not use a single model but a set of models trained on the same data. Each trained model generates a slightly different prediction due to its uncertainty in the architecture, such as the regularisation techniques used, which allows us to assess better the model’s accuracy and the robustness of the results when applying different collision detection techniques.

In this study, the deep learning models are the foundation for predicting vessel trajectories, which are then used to assess collision risks. Each model generates a probabilistic forecast of the vessel’s future positions, accounting for uncertainties inherent in maritime navigation. The predicted trajectories are further analysed using statistical methods such as PIs, CIs, EPRs, and CPRs to establish boundaries for possible collision zones. We evaluate the likelihood of a collision by comparing these predicted boundary widths with the actual positions of vessels. This integrated approach ensures that the collision risk assessment is closely tied to the accuracy of the trajectory predictions, providing a dynamic and probabilistic evaluation of potential collision scenarios.

### 3.2. Confidence and Prediction Intervals

Confidence and prediction intervals are statistical measures that are used to quantify uncertainty in predictions, particularly for models that output numerical values, such as those predicting vessel trajectories. Both intervals often rely on assumptions about data distribution, for example, a normal distribution, and are typically derived from theoretical distributions. CIs estimate the range within which a population parameter (e.g., mean) is expected to lie with a certain confidence level [45]. PIs provide a range within which we can expect future data points to fall with a certain confidence level, α, typically 95%. $W_{\mathrm{PI}}$ is wider than $W_{\mathrm{CI}}$ because it accounts for the individual variability of each data point around the predicted mean, not just the variability of the mean itself [46]. In our scenario, the intervals for each time step are computed individually by aggregating point data from various models that forecast the same sequence. This approach ensures that the intervals accurately reflect the range of predictions and the associated uncertainty at each specific moment, thereby accommodating the variability inherent in the models’ outputs. Given a set of points at a given time containing the predicted coordinates, the CI (2) and the PI (1) at the confidence level α are calculated using the equations where the upper and lower bounds are subsequently found by adding/subtracting the sums from the sample mean and formula expressions based on [47,48,49]:

(1) $$\begin{array}{cc} W_{\mathrm{PI}} & =t_{\left(\frac{1+\alpha }{2},n-1\right)}\cdot SD\cdot \sqrt{1+\frac{1}{n}}, \end{array}$$

(2) $$\begin{array}{cc} W_{\mathrm{CI}} & =t_{\left(\frac{1+\alpha }{2},n-1\right)}\cdot \frac{SD}{\sqrt{n}}, \end{array}$$

where the following applies:

- $t_{\frac{1+\alpha }{2},n-1}$ is the critical value of the student’s distribution (*t*-score) corresponding to the desired confidence level, α, and n−1 degrees of freedom, where *n* is the sample size.
- SD is the sample’s standard deviation, representing the spread of the data points.
- $\frac{SD}{\sqrt{n}}$ is the standard error of the estimate, which adjusts the standard deviation for the size of the sample.
- The term $\sqrt{1+\frac{1}{n}}$ accounts for the added uncertainty when predicting a single future observation rather than estimating the population’s mean.

In standard univariate linear regression models, statistical intervals are delineated and visualised in a relatively uncomplicated manner, typically with time series data plotted along the x-axis and any other variable of interest on the y-axis. However, in our scenario, the forecast data are multivariate, encompassing longitude, latitude, and time series. For bi-variate coordinate data, these intervals are often represented as an ellipse around the mean of the data points, capturing the uncertainty in both dimensions. Here, the intervals serve as the ellipse’s radii. This approach is utilised to define the domain of the ship’s trajectory, thereby accommodating the multidimensional nature of the data.

### 3.3. Ellipsoidal Prediction Regions

Unlike univariate data, which contain a single variable, multivariate data contain multiple variables that can be related to each other. For example, in geographic information systems (GISs), coordinates such as latitude and longitude are often analysed together. In vessel trajectory prediction, quantifying the uncertainty of the prediction is as important as the prediction itself. For this purpose, the EPR is constructed to encapsulate the potential future position of the vessel within a confidence range. EPR is mathematically formulated (3) by considering the predicted trajectory points’ variance and spatial distribution based on the author’s formulation [18]. The axes of the ellipsoid correspond to the principal directions of variability in the data, and their lengths are proportional to the standard deviations of the data along these directions. For the expression of data points with mean vector μ and covariance matrix Σ, an EPR that contains a 100(α)% portion of the distribution can be defined as

(3) $$EPR=\{x:{\left(x-\mu \right)}^{T}\Sigma^{-1}\left(x-\mu \right)\leq \chi_{p,\alpha }^{2}\},$$

where *x* is a vector in the multivariate space, $\chi_{p,\alpha }^{2}$ is the critical value of the chi-squared distribution with *p* degrees of freedom corresponding to the desired confidence level α, and *p* is the number of variables. Given a set of predicted points for a vessel’s trajectory and a centre point, the EPR can be calculated using the provided pseudo-code, which the authors of the article produce.

First, the convex hull surrounding all points is determined to find the outer boundary. The covariance matrix is then derived, and eigenvalue decomposition is performed to extract eigenvalues and eigenvectors. The chosen confidence level (represented by α) is set to 0.95 (corresponding to 95%). The radius of the EPR ellipsoid is calculated using the chi-square distribution, considering α and the degrees of freedom, which, for a 2D point, are equal to 2, resulting in the radius $\sqrt{\chi_{2,\alpha }^{2}}\cdot \sqrt{\lambda_{i}}$. Angles ranging from 0 to 2π are then generated to represent the ellipsoid parametrically. The surface points of the ellipsoid are calculated using these radii and then rotated by the eigenvectors to align the ellipsoid with the data distribution. Finally, an ellipsoid is formed from the centre point, creating the EPR (see Algorithm 1).

EPRs have basic geometric characteristics and are classified as parametric methods. This classification arises from the reliance on distribution parameters such as shape, orientation, and size to define the domain. Nonetheless, these domains of the predictions are particularly valuable for the analysis of multidimensional ship orbital collision data. Their usefulness becomes apparent when the study focuses on understanding the ships’ geographical position and spatial extent.
**Algorithm 1** EPR calculation.    **function** CalculateEPR(points, center)
          **Input:**
points—array of latitude and longitude points
          **Input:** center—center point (mean) of the EPR          **Output:**
epr—the calculated ellipsoidal prediction region as a polygon           *hull*←ConvexHull(*points*)          hullVertices←points[hull.vertices]          *covMatrix*←Covariance(*hullVertices*)          [*eigValues,eigVectors*]←Eigen(*covMatrix)*)          confidenceLevel←0.95          $radii\leftarrow \sqrt{C\mathrm{HI}S\mathrm{QUARED}I\mathrm{NVERSE}(confidenceLevel,2)}*\sqrt{eigValues}$           angleArray←arrayofanglesfrom0to2π          ellipsoidPoints←EMPTYARRAY(size:100,dimensions:2)          **for** i←1 **to** 100 **do**              ellipsoidPoints[i][1]←radii[1]∗cos(angleArray[i])              ellipsoidPoints[i][2]←radii[2]∗sin(angleArray[i])          **end for**          rotatedPoints←eigVectors·ellipsoidPoints           translatedPoints←rotatedPoints+center          epr←CREATEPOLYGON(translatedPoints)          **return** epr    **end function**


### 3.4. Conformal Prediction Regions

Conformal prediction intervals aim to provide a range (or region, in the case of multidimensional predictions) within which future observations are expected to fall, with a predefined level of confidence or significance, using past data [50,51]. CPR leverages the past distribution of data to define prediction regions guaranteed to contain the true value of new observations with a specified probability, assuming that future data will resemble the past data.

For CPR, a calibration set C, consisting of data instances with known true outcomes, is employed to calculate nonconformity measures, denoted as N. This calibration set is distinct and non-overlapping with the model’s training and test sets, ensuring the utilisation of validation samples previously employed to ascertain the model’s accuracy during its training phase.

In the CPR, a calibration set, C, consisting of data instances with known true outcomes, is employed to calculate nonconformity measures, denoted as N. This calibration set is distinct and non-overlapping with the model’s training and test sets, ensuring the utilisation of validation samples previously employed to ascertain the model’s accuracy during its training phase.

We use $Y_{t}$ to denote the actual multivariate coordinates at time step *t* and ${\hat{Y}}_{t}$ to denote the predicted coordinates. The nonconformity measure for each time step is quantified using the Euclidean distance (4):

(4) $$N_{t}\left(Y_{t},{\hat{Y}}_{t}\right)={∥Y_{t}-{\hat{Y}}_{t}∥}_{2}$$

where t∈{1,…,T} indexes the time steps within the sequence. The Euclidean distance is a natural choice here but is suitable for determining the width of a region. The nonconformity measure should account for errors in all dimensions of the multivariate output, including latitude and longitude in the case of spatial coordinates.

Considering the tendency for prediction errors to increase over longer forecast horizons, the nonconformity scores are not aggregated over the entire sequence. Instead, they are averaged at each time step, resulting in a distinct threshold, $\tau_{t}$, for each time step. This threshold is subsequently applied to new prediction sequences from the test sample to determine if each predicted point is within the expected conformal region. To ensure an unbiased evaluation, the calibration dataset comprises 15% of the full dataset, specifically selected from the validation set. This dataset is distinct from the training data used for model building and the test data used for evaluating final model performance. The model generates predictions for each instance using this calibration set, and the corresponding nonconformity scores are calculated. In the multi-model approach, these scores represent an average of the distances across all models at each time step. The problem of calibrating CPR is approached by calculating the average nonconformity scores across all models at each time step in the calibration set. For the *i*-th instance in the calibration set and time step *t*, the nonconformity score $R_{t}^{\left(i\right)}$ is defined as the Euclidean distance between the actual value, $Y_{t}^{\left(i\right)}$, and the predicted value, ${\hat{Y}}_{t}^{\left(i\right)}$.

The average nonconformity score at time step *t* across all instances is obtained using (5):

(5) $${\bar{R}}_{t}=\frac{1}{n}\sum_{i=1}^{n}R_{t}^{\left(i\right)},$$

where *n* denotes the number of instances in the calibration set. Consequently, we seek to optimise the following problem (6):

(6) $$\begin{array}{cc} \mathrm{minimize}\; & \mathrm{Quantile}\left(\{{\bar{R}}_{1},\ldots ,{\bar{R}}_{T}\},1-\delta \right)\\ \mathrm{subject}\;\mathrm{to}\; & \sum_{t=1}^{T}\alpha_{t}{\bar{R}}_{t}=1\\ \; & \alpha_{t}>0,\;t=1,\ldots ,T \end{array}$$

Here, $\alpha_{t}$ represents the parameters to be optimised, which scale the nonconformity scores at each time step, and δ represents the desired confidence level for CPR. The objective function minimises the quantile of the averaged nonconformity scores, aligning with the confidence level to construct valid PIs.

**Figure 3:**
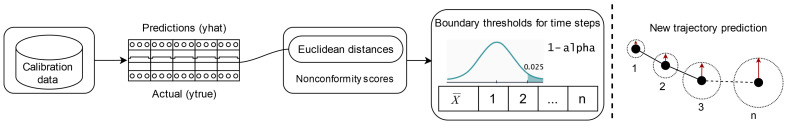
Illustration of boundary width determination in CPR using nonconformity scores.

Figure 3 illustrates the process, from using the calibration data to obtaining the new trajectory prediction with uncertainty estimation. The calibration data sequences, which contain both the input and output parts (as part of the supervised learning process), are used as input for the trained models to generate predictions ($\hat{y}$) over multiple time steps. These predictions are then compared to the corresponding ground truth values ($y_{\mathrm{true}}$) from the calibration set output to calculate nonconformity scores based on the Euclidean distances between the predicted and actual values. These nonconformity scores are then used to compute boundary thresholds for each time step, determined by the confidence level 1−α. These thresholds define the prediction region width, representing the outlying distance radius, illustrated as circles around each future predicted position in the new trajectory. This ensures that the true position lies within the defined regions with a given confidence level. The region’s width for new predictions, which is consistent with the desired confidence level, is established by this quantile, allowing for an assessment of the accuracy of the forecasts. This method is more empirical, constructed from the data without strong parametric assumptions, making them widely applicable.

## 4. Experiments

In this study focused on predicting vessel trajectories, we adopt an extensive methodology that synergises advanced deep learning models with robust statistical methods. This integrated approach is designed not only to forecast the future paths of maritime vessels but also to meticulously quantify the uncertainty inherent in such predictions. By doing so, the study provides a trajectory estimate and a nuanced probabilistic understanding of potential navigational outcomes, enhancing the reliability and application of these predictions in maritime operations. The experiments are conducted using an NVIDIA RTX 3080 GPU board and the VU institute HPC cluster to ensure computational efficiency. The implementation is performed using Python (version 3.9.16) with the Spyder 5.4.3 IDE on a Windows 10 operating system. The primary libraries utilised for data processing and model training include NumPy (1.24.3), PyProj (3.6.0) (Proj, Geod), Keras (2.10.0), and TensorFlow (2.10.1).

### 4.1. Data and Experiment Set-Up

**Figure 4:**
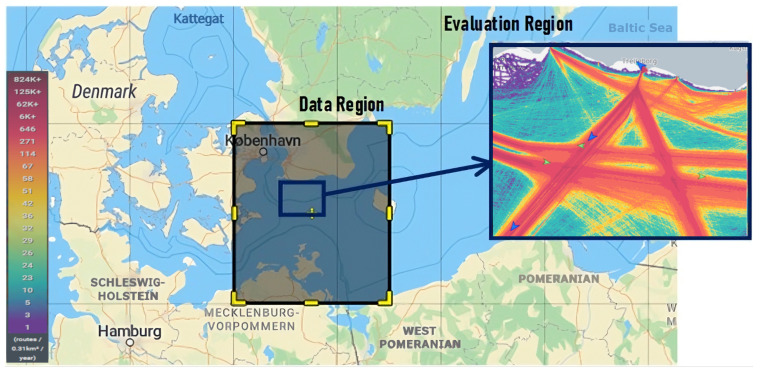
AIS study region with highlighted evaluation area.

The LSTM autoencoder model from the Baltic Sea was developed using six months of AIS data (http://web.ais.dk/aisdata/, accessed on 3 June 2024). The dataset covering June 2021 to December 2021 includes information from various ship types, such as cargo, passenger, fishing, and other vessels, with input and output sequences of the same length formulated as matrices. The geographical scope of the data collection is shown in Figure 4, where the boundary box coordinates are the following: west = 12′00°, east = 15′00°, north = 56′00°, and south = 54′00°, covering almost 950,000 sequences. These sequences are then divided into sets as described in Section 3. On average, the vessel travels about 16 kilometres per sequence, almost 320 m per minute (per time step), depending on the vessel type. The regions used for simulated test case predictions are delineated in smaller subsets of the data, called evaluation regions in the figure. This subset is chosen due to its high traffic density and proximity to ports. The study methods are evaluated in this particular sample, although predictions could also be made using other sequences over a wider range of data regions.

**Figure 5:**
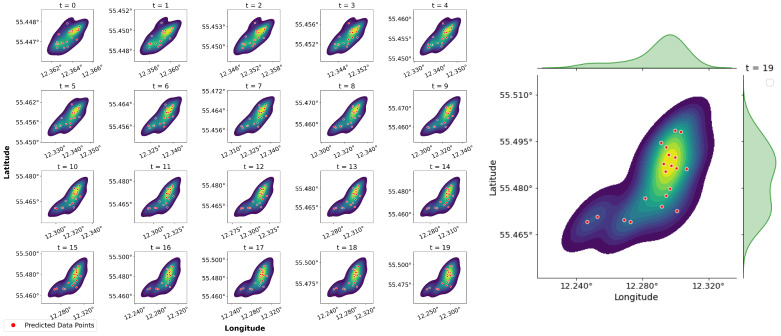
Distribution of data points for selected sequence predictions.

Predictions must be made for each sequence using a different model to construct and evaluate the prediction regions. Figure 5 shows randomly selected sequences and their trajectory predictions generated by the 20 models. Each subplot in the figure represents a density estimate or distribution of the predicted points at a given time. Areas with higher density around the geometrical centre are shown in warmer colours (yellow tones), and areas with lower probability are shown in cooler colours (blue tones). Actual positions are marked with red dots corresponding to the 20 min further predictions. The plot indicates that the data do not exhibit a perfectly normal distribution as evidenced by the absence of a single distinct peak. This characteristic poses limitations for using parametric methods such as PI and CI, which rely on assumptions of normality. However, forecast points are generally centralised, especially early in the forecast period. This tendency to focus on the early stages of forecasting is common in predictive analytics.

### 4.2. Region Evaluation

The estimated area of a region or width is used for the standard assessment. The evaluation of the sequences within the sub-region, totalling 8984, and their corresponding prediction regions hinges on two principal criteria: (a) an examination of whether the actual trajectory of the vessel at each time step resides within the predicted region, a method known as coverage probability; and (b) the verification of the methods’ effectiveness in a real marine traffic incident through the calculation of probabilistic risk score of collision. All methods used correspond to a 95% confidence level.

Let $A=\{a_{1},a_{2},\ldots ,a_{n}\}$ represent the set of actual points, where $a_{i}$ is the actual coordinate at time step *i*, and let $Z=\{z_{1},z_{2},\ldots ,z_{n}\}$ represent the set of predicted zones, where $z_{i}$ is the predicted zone at time step *i*. We define the indicator function (7) *I* for each time step *i* as follows:

(7) $$I\left(a_{i},z_{i}\right)=\left\{\begin{array}{cc} 1 & \mathrm{if}\;\mathrm{point}\;a_{i}\;\mathrm{is}\;\mathrm{contained}\;\mathrm{within}\;\mathrm{zone}\;z_{i},\\ 0 & \mathrm{otherwise}. \end{array}\right.$$

The score for the full set of sequences, assessing the predicted zones’ suitability for the actual points, is the sum of the individual scores over all time steps (8), where *n* is the total number of time steps or points. The accuracy of the predicted zones can then be expressed as a percentage (9). This accuracy metric shows the percentage of time steps where the actual coordinates fall within the predicted intervals.

(8) $$\begin{array}{cc} \mathrm{Score} & =\sum_{i=1}^{n}I\left(a_{i},z_{i}\right), \end{array}$$

(9) $$\begin{array}{cc} \mathrm{Accuracy} & =\left(\frac{\mathrm{Score}}{n}\right)\times 100\%. \end{array}$$

To calculate the probability of intersection, for example, between the predicted trajectories of ships (which are represented as ellipses/circles within prediction zones), we use the following Formula (10):

(10) $$P\left(\mathrm{collision}\right)=\frac{V_{A\cap B}}{V_{A}+V_{B}-V_{A\cap B}},$$

where $V_{A}$ and $V_{B}$ are the areas of the individual regions, and $V_{A\cap B}$ is the area of their intersection. The formula considers the areas of both zones and the overlapping area. If the two regions do not intersect, the overlapping area will be zero, resulting in a zero probability of intersection.

The idea is related to a classical probability formula, where the probability of an event *C* happening is the number of outcomes that result in *C* divided by the total number of possible outcomes. We assess the likelihood of ships intersecting based on their projected paths within their respective prediction zones.

### 4.3. Verification: Collision Between Scot Carrier and Karin Hoej Cargo Ships

On 13 December 2021, a critical maritime incident occurred in the Baltic Sea involving a collision between two cargo ships. The event unfolded off the coast of the southern Swedish city of Ystad and near the Danish island of Bornholm, within Swedish territorial waters. This collision involved a Danish-flagged vessel, Karin Hoej (MMSI: 219021240), and a British cargo ship, the latter identified as the Scot Carrier (MMSI: 232018267). In the aftermath of the collision, the Danish ship capsized, leading to an immediate and urgent search and rescue operation for the crew members aboard. The search efforts, intensified by the participation of the Scot Carrier, focused on locating at least two people initially reported missing. Tragically, the situation took a sombre turn when one of the missing crew members from the Danish vessel was found dead in the hull, highlighting the severe consequences of the incident. The collision, amidst fog and darkness, has prompted a comprehensive investigation into the circumstances leading to this unfortunate event, examining factors such as maritime traffic negligence and the adherence to safety protocols in one of Europe’s busiest maritime corridors.

**Figure 6:**
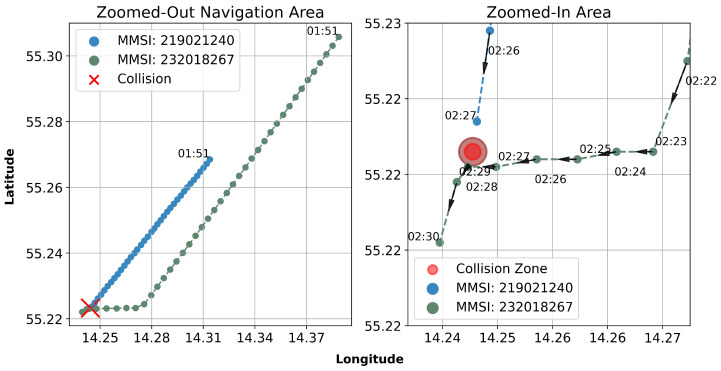
Spatio-temporal analysis of maritime collision accident.

Figure 6 presents two scatter plots that delineate the spatio-temporal trajectories of two vessels before and immediately following a collision in the Baltic Sea. The data, extracted from the Danish Maritime Authority’s AIS, offer a high-resolution depiction of the incident, which occurred around 2:27 a.m. at night time. The ‘Zoomed-Out Area’ on the left illustrates the direction paths of the parallel moving vessels identified by MMSI 219021240 (blue) and MMSI 232018267 (green) before their collision (timestamp 1:51). The adjacent ‘Zoomed-In Area’ on the right provides a detailed view of the vessel movements at one-minute intervals from 02:22 to 02:30, showing the collision zone and directions of movement. This granular temporal resolution reveals the immediate navigational circumstances that led to the collision. Following the collision, the AIS signal transmission from Karin Hoej was disrupted, ceasing data flow to the information system.

This visual analysis is crucial for the forensic examination of the collision’s causes and consequences. It precisely demonstrates the trajectories and relative positions of the vessels, highlighting the importance of AIS data in maritime safety investigations. The figure not only contributes to elucidating this particular incident but also exemplifies the significance of AIS data from maritime authorities for enhancing navigational safety protocols and collision avoidance strategies.

**Figure 7:**
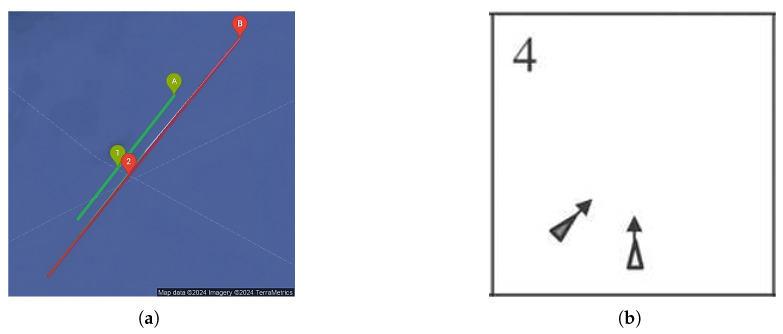
Graphs of collision risk assessment: (**a**) calculation of CPA and TCPA for cargo vessels half an hour before collision; (**b**) Imazu problem case 4 schematic representation (extract from the source [52]).

According to the COLREGs, the captain of a vessel on the open sea should maintain a minimum distance of 1.5 to 2 nautical miles from other vessels to avoid dangerous situations. One of the tools used in maritime and aviation contexts is the CPA, which represents the shortest distance between two moving objects if they continue on their current course and speed without any changes. Meanwhile, the TCPA measures the time until two moving objects reach the CPA to each other. These methods are applied to the collision verification for this study to utilise existing tools. Measurements are made based on the position of the vessels at 01:51 timestamp as shown in the figures. The geographical position (longitude and latitude), speed in knots, bearing, and angles in degrees are used for the CPA calculation. The resulting CPA is 0.53 nautical miles, indicating an increased risk since the distance is less than 1 nautical mile. The TCPA is calculated as 34.80 min to the calculated CPA points. Figure 7a illustrates the results of the calculations, indicating the starting point of the calculation with letters, the lines representing direction, and the projected position points with numbers. In maritime navigation, collision avoidance scenarios can be analysed using established theoretical models such as the Imazu problem, which provides a framework for evaluating vessel manoeuvring strategies to minimise collision risks. Our real-case scenario closely aligns with the Imazu problem case 4 (see Figure 7b), where the relative positions and courses of two vessels necessitate a careful assessment of collision avoidance measures [52].

**Figure 8:**
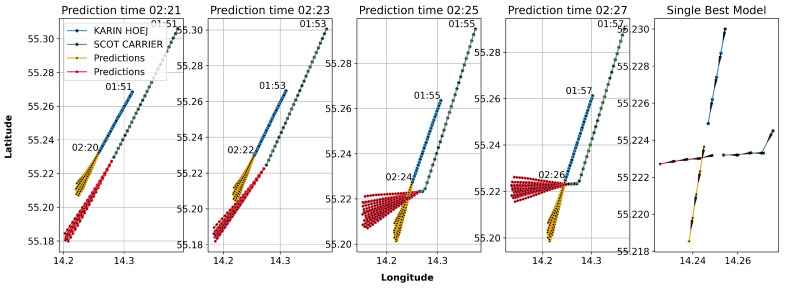
Comparative trajectory predictions from deep learning models. Triangles in the ‘Single Best Model’ subplot indicate the vessel’s moving direction.

Figure 8 displays a series of scatter plots juxtaposing actual and predicted vessel trajectories leading up to the maritime collision near Bornholm, as deep learning models forecasted. Each panel represents a different prediction start time, ranging from 02:21 to 02:27, with a 30-step input sequence utilised for forecasting the future positions of the vessels. The plots depict the actual paths of the KARIN HOEJ (blue) and the SCOT CARRIER (green) and overlay these with the predicted trajectories (yellow and red) generated by our trained models at each interval. The architecture of the deep learning autoencoder, with its inherent complexities and the incorporation of regularisation techniques such as Dropout layers, results in the model producing a range of predictions with subtle variations. The rightmost graph, titled ‘Single Best Model’, showcases the most precise model forecasted trajectory, identified by its minimal error value. Additionally, all depicted paths include directional indicators.

This ensemble of predictions illustrates the capabilities of our deep learning models to anticipate vessel movements and their potential for application in real-world maritime navigation and safety systems. It also underscores the value of employing a suite of models to capture the variability and uncertainty inherent in such dynamic prediction scenarios.

**Figure 9:**
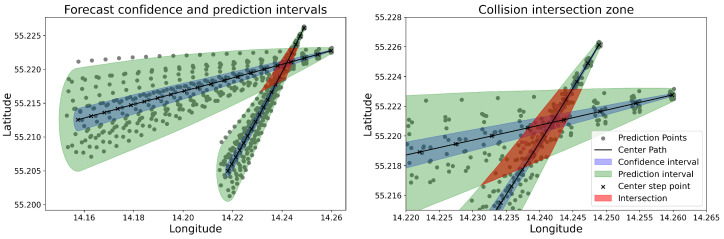
Visualisation of prediction and confidence intersection zones for bi-variate spatial data.

Predictive calculations for the ship trajectories are initiated from the 2:25 timestamp, coinciding with observing the ships’ turning movement changes. However, the starting point for predictions can be chosen at any time. Figure 9 comprises two panels illustrating the use of CIs in predicting bi-variate spatial data for maritime trajectory forecasting. The left panel, ‘Forecast Confidence and Prediction Intervals’, depicts the calculated CIs for a 20 min (steps) prediction horizon determined by our trained model. For each step, the data are represented by a dot, aligning within the shaded confidence interval bands, demonstrating the range of potential vessel locations over time. The centre of the predicted path is marked by cross symbols, which indicates the model’s central forecast for the vessel’s position at each time step. A red zone marks the intersection area, representing the forecasted convergence of the two vessel paths. The graphical representation of the forecasts is made by interpolating the data between the time series, as the prediction regions are normally represented as circular, elliptical or selector ship domains.

The right panel, named the ‘Collision Intersection Zone’, zooms in on the area where the CIs of the two predicted trajectories overlap. This intersection signifies a heightened risk of collision as indicated by the narrowing intervals in the initial prediction steps. The darkest shaded area denotes the most immediate risk zone, based on the closest predictions where the paths are forecasted to intersect. The reliability of both intervals is 95%. The desired level of confidence decreases, the range of the interval decreases (becomes narrower), and as the confidence level increases, the range of the interval increases (becomes wider).

Together, these panels convey the dynamic nature of predictive modelling in maritime navigation, emphasising the importance of calculating and visualising the predictions and associated CIs to assess the risk of collision more effectively. The models demonstrated a high degree of accuracy in forecasting the collision, which do occur.

**Figure 10:**
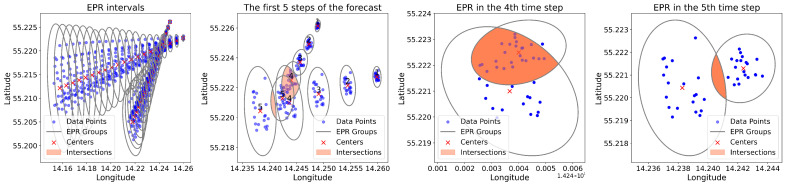
Ellipsoidal prediction regions. Numbers next to the red crosses represent the corresponding prediction time steps.

Figure 10 presents a graphical analysis of vessel trajectory forecasts using the EPR method. It consists of four distinct panels, each illustrating the application of EPRs at different stages of the prediction process.

In the first panel, labelled “EPR intervals”, a series of ellipses represent the EPRs for multiple forecasted time steps of two vessels. The data points (blue and red dots) indicate the actual positions of the vessels. At the same time, the ellipses encapsulate the regions where the vessels are predicted to be with a certain level of confidence. The ellipses oscillate, demonstrating the dynamic nature of the prediction over time, with overlapping areas suggesting potential collision risks.

The second panel, “The first 5 steps of the forecast”, zooms in on the initial predictions. It shows a closer view of the individual EPR ellipses for the first five time steps, where intersections of the ellipses (black outlines) indicate moments where the prediction models suggest a higher likelihood of a collision. The blue and red dots continue to represent the actual positions of the vessels, and the ‘X’ marks denote the centres of the EPR ellipses, which are the model’s best guess for the vessels’ future positions.

In the third and fourth panels, “EPR in the 4th time step” and “EPR in the 5th time step”, we see isolated snapshots of the EPRs at specific moments. These individual ellipses provide a clear visual representation of the predicted movement areas for each vessel at given time steps. The overlap between the ellipses is shaded, highlighting the critical areas where the vessels’ paths are predicted to converge, indicating moments of heightened collision risk.

By leveraging a multi-model approach, we effectively quantify prediction variability, where uncertainty increases as the forecast horizon extends. This is directly reflected in the size of the ellipsoidal boundaries, as longer-term predictions exhibit greater divergence due to accumulating error. This characteristic is inherent in stochastic trajectory forecasting, where uncertainty propagation is a fundamental aspect of risk assessment. The probabilistic nature of EPRs allows for a more robust evaluation of vessel movement safety compared to single deterministic methods such as CPA, which focus on immediate proximity rather than future trajectory regions.

### 4.4. Analysis of Prediction Accuracy and Risk Estimation

In this subsection, we present the evaluation of the model’s performance based on test set predictions. The analysis includes the average prediction error according to time steps, offering insight into the accuracy of the model’s forecasts. Additionally, we assess the coverage probability, determining how often the actual vessel positions fall within the predicted collision regions. Finally, we verify the proposed methods using a real-world case study involving the Scot Carrier and Karin Hoej cargo ships. In this analysis, we calculate the predicted ship domain boundaries based on future vessel positions using different methods presented in the research. By evaluating the area of overlap between these boundaries, we assess the probability of a potential collision, providing a practical assessment of the model’s effectiveness in forecasting maritime safety risks.

**Figure 11:**
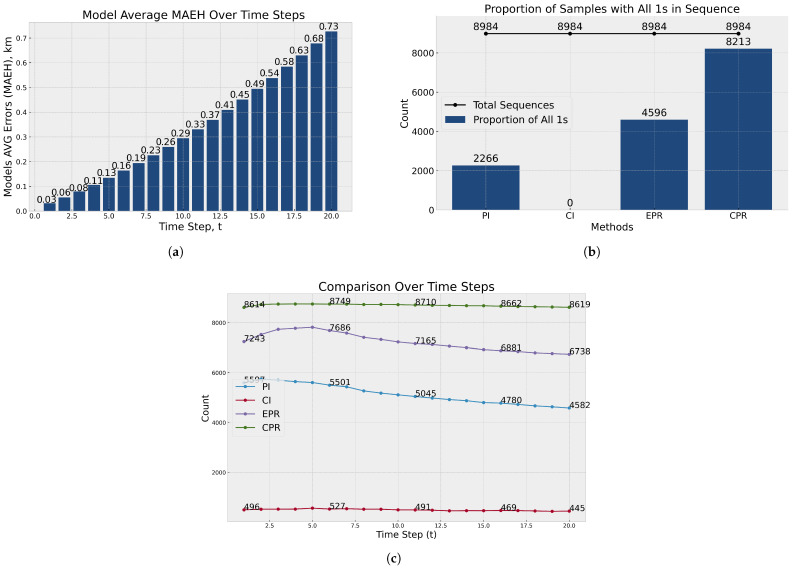
Graphs of accuracy and evaluation results: (**a**) average model error by time series of trajectory prediction (km); (**b**) sequences with all time steps within the calculated boundary widths (coverage probability) estimation; and (**c**) coverage probability count across individual time steps.

The accuracy of each model trajectory prediction is typically gauged using regression metrics such as Mean Squared Error (MSE), Mean Absolute Error (MAE), and Mean Absolute Percentage Error (MAPE), among others. In our study, we assess the model’s accuracy utilising a derived Mean Haversine Absolute Error function (MAEH), which aligns conceptually with MAE but calculates the discrepancy based on the distance between the time series of the prediction and the actual sequence rather than direct coordinate comparisons [6]. This approach quantifies the distance error in the International System of Units (SI) as meters or kilometres. Figure 11a depicts the cumulative average prediction error across all models. The error fluctuates between 30 and 730 m for forecasts extending up to 20 min, increasing as the forecast timeline extends. This metric’s evaluation is conducted on a designated test sample set (15% of all data sequences).

Figure 11b assesses the subset sequences following the scoring methodology outlined previously (8), with an exception: this graph displays the aggregate count of sequences accurately predicted by the methods, specifically those instances where all the time series points within a sequence are contained within the predicted region. It reveals that most predictions align with the CPR areas, indicating high accuracy. Conversely, the confidence interval—though set at a 95% confidence level—is markedly narrow, resulting in the absence of sequences fully encompassed within these intervals. Figure 11c illustrates the distribution of sequence entries in the forecast region across time series and the applied method. It shows that as the forecast horizon increases, the accuracy of EPR and the prediction and confidence intervals tend to decrease. In contrast, CPR areas are established and calculated using the calibration sample, featuring a broader radius. This characteristic increases the likelihood of points falling within the CPR, enhancing its inclusively. However, it is important to note that this method relies on empirical data from past observations rather than a theoretical probabilistic assumption.

**Figure 12:**
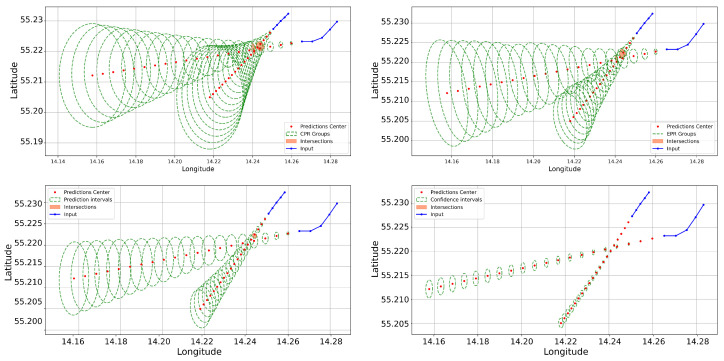
Comparison of method boundary widths in a marine accident.

Visualisation of the regions as circular or elliptical vessel guard zones (domains) is feasible if the semi-major and semi-minor axes are known or, at the very least, the radius area. For instance, in the context of EPR, where both the minor and major axes are specified, the representation takes the form of an ellipse. Conversely, the representation defaults to a circle for the CPR, where only the radius is determined—indicating the maximum Euclidean distance a point can deviate from the centre. Figure 12 exhibits the marine incident case previously discussed, showcasing various implementations of these methods (without resorting to additional interpolations purely for visualisation purposes).

Once an area is demarcated, its possible overlap with adjacent areas can be analysed by predicting ship trajectories. To make it easier to understand the area covered by each zone, they are converted to a UTM (Universal Transverse Mercator) projection, which allows the actual area of these zones to be calculated in square meters.

Applying the Formula (10) can obtain a probability estimate of the collision risk score. The data presented in Table 2 show that using the CPR method results in a risk of collision between two ships within a 5 min time frame of nearly 40%, as predicted 02:24. Conversely, even if the risk of a collision is 1%, i.e., an incident is likely to happen, it is much less likely. Zone A is Karin Hoej, and Zone B is Scot Carrier. The intersection areas between the trajectories of different ships at a specific point in the time series, for instance, at step 1, are examined by comparing them with the forecast trajectory of the next ship at the same step, and this process continues sequentially. The centres of the predicted trajectories for all models are marked with red dots. This methodical approach ensures a thorough evaluation of the potential overlaps or intersections among the predicted paths of various vessels, providing insight into the likelihood of close encounters or collisions at each step of the forecast.

## 5. Discussion and Conclusions

This study demonstrates that integrating multiple models with advanced statistical and geometrical methods enhances the accuracy of assessing uncertainty in vessel trajectory predictions and their applications in maritime collision detection. Methods such as ellipsoidal prediction regions, prediction intervals, confidence intervals, and conformal prediction regions provide a comprehensive framework for defining trajectory prediction boundaries, often referred to as guard zones or ship domains. This integration allows for a robust and precise assessment of collision risks, surpassing traditional methods such as CPA and TCPA.

One of the key contributions of this research lies in using multiple models to assess trajectory prediction uncertainty without relying on traditional bootstrapping techniques. By leveraging variations across models, the approach captures a more comprehensive range of possible outcomes, contributing to reliable boundary determination and improving the robustness of collision detection. Furthermore, a unique method for calculating and evaluating overlapping trajectory prediction boundaries from each vessel’s perspective is proposed. This probabilistic measure of collision risk, expressed as a collision risk score using a Jaccard index, offers a significant advantage over traditional deterministic approaches by considering the entire trajectory prediction boundary.

The practical applicability of the framework is validated using a real-world case study: the 2021 collision between the Scot Carrier and Karin Hoej cargo vessels. This validation highlights the utility of the proposed methods in detecting potential collisions and demonstrates their effectiveness in identifying overlapping boundaries and assessing collision risk. Conformal regions prove particularly effective among the methods, achieving a 39.6% collision risk score in the real-world scenario at a 95% confidence level. This underscores the potential of nonconformity for improving collision detection and maritime safety in combination with trajectory predictions.

While these findings demonstrate the potential of the proposed framework, several limitations should be acknowledged. The methods depend on the accuracy of trajectory predictions, which are influenced by the quality of the input data. AIS data, while comprehensive, may contain inconsistencies such as signal gaps or delays. Resampling techniques are employed to address these issues, but further refinement in data preprocessing is necessary to ensure uniformity. Additionally, the current approach assumes a certain degree of exchangeability in the data and does not yet include external factors such as meteorological conditions or vessel-specific physical parameters. Incorporating these factors in future work could enhance the precision and applicability of trajectory predictions.

Furthermore, we incorporate CPA/TCPA as a conceptual benchmark, recognising its widespread use in real-time collision assessment. However, CPA is a deterministic method that does not account for uncertainty in vessel movements. Our approach evaluates clusters of uncertainty and extends beyond the CPA single-point analysis to provide a more robust framework for assessing collision probabilities. The 20 min prediction window corresponds to the stopping times of large vessels, effectively serving as our TCPA (time to collision) threshold for detecting potential collision risks. Unlike CPA, which determines a single deterministic point within a guard zone at a specific position, our method evaluates probabilistic trajectory boundaries, offering a broader assessment of future vessel movements. To trigger an alarm, CPA requires predefined proximity (e.g., 0.25 NM) and response time (e.g., 15 min) thresholds. In contrast, our approach adopts a probabilistic perspective by forecasting future trajectory boundaries and analysing their overlap, enabling a more dynamic and comprehensive evaluation of collision risks. However, this also introduces computational challenges, particularly in real-time applications, given the resource-intensive nature of training multiple models.

Looking ahead, this framework offers significant potential for expansion. Future research could integrate physical vessel parameters, such as length and beam, and meteorological data to enhance prediction accuracy. Exploring comparative studies with alternative state-of-the-art methods, including heuristic and reinforcement learning-based approaches, would provide a broader benchmark for evaluation. Additionally, integrating this framework into real-time maritime systems, such as autonomous navigation or anomaly detection platforms, could unlock further applications to ensure maritime safety.

After addressing these limitations and building on the demonstrated strengths, the proposed methods present a promising step forward in maritime collision detection and risk assessment, contributing to safer and more reliable vessel navigation systems.

## Figures and Tables

**Table 1:** LSTM autoencoder hyperparameter configuration.

Parameter	Value	Note	Parameter	Value	Note
Layers	3	Total number of encoder and decoder parts	Number of Units	275	Cells in each LSTM layer
Seq. Len.	50	30 input, 20 output	Batch Size	128	Examples utilised in one iteration
Epochs	100	-	Models Size	20	Models trained on the same data
Optimiser	0.001	Adam (with Learning Rate)	Loss F-ion	MSE	Measures prediction quality
Regularisation	0.01	Dropout layers	Activation F-ion	ReLU	Used in LSTM gates and Dense

**Table 2:** Collision risk score comparison over time steps (highest probability marked in bold).

Time Step	EPR				CPR			
	Area A, m^2^	Area B, m^2^	A∩B, m^2^	Prob (%)	Area A, m^2^	Area B, m^2^	A∩B, m^2^	Prob (%)
1	11,425.5	32,978.4	0.0	0.00	20,812.7	20,808.5	0.0	0.00
2	35,960.2	117,517.0	0.0	0.00	64,937.4	64,927.8	0.0	0.00
3	72,216.4	220,926.0	0.0	0.00	153,537.9	153,523.8	0.0	0.00
4	132,867.0	368,967.0	96,986.7	23.96	302,596.1	302,584.9	171,576.5	**39.57**
5	198,320.0	536,512.0	22,066.5	3.10	524,334.6	524,344.3	89,920.4	9.38
6	274,578.0	762,444.0	0.0	0.00	826,551.7	826,611.9	0.0	0.00
Time Step	PIs				CIs			
	Area A, m^2^	Area B, m^2^	A∩B, m^2^	Prob (%)	Area A, m^2^	Area B, m^2^	A∩B, m^2^	Prob (%)
1	4717.5	14,151.0	0.0	0.00	224.6	673.9	0.0	0.00
2	14,568.8	53,199.1	0.0	0.00	693.8	2533.3	0.0	0.00
3	31,642.8	112,766.8	0.0	0.00	1506.8	5369.8	0.0	0.00
4	53,492.5	190,868.5	8420.9	3.57	2547.3	9089.0	0.0	0.00
5	79,293.0	288,027.5	0.0	0.00	3775.9	13,715.6	0.0	0.00
6	107,950.1	405,907.8	0.0	0.00	5140.5	19,328.9	0.0	0.00

## Data Availability

The source code used in this study is available at the public repository https://github.com/RobJur/rnn-vessels-trajectory-prediction (accessed on 20 February 2025), which includes the implementation of the research methods, LSTM autoencoder architecture, and data preprocessing steps. The AIS data utilised in this study were obtained from the Danish Maritime Service, Safety of Navigation, National Waters, Caspar Brands Plads 9, 4220 Korsør, Denmark. The data are publicly accessible online at http://web.ais.dk/aisdata/, (accessed on 3 June 2024) and pertain to the Baltic Sea region.

## Abbreviations

The following abbreviations are used in this manuscript:

AIS	Automatic Identification System
CI	Confidence interval
CPA	Closest Point of Approach
CPR	Conformal prediction region
EPR	Ellipsoidal prediction region
HELCOM	Helsinki Commission
LSTM	Long Short-Term Memory
MAEH	Mean Haversine Absolute Error
MMSI	Maritime Mobile Service Identity
PI	Prediction interval
TCPA	Time to Closest Point of Approach
